# The Impact of Environmental Factors in Influencing Epigenetics Related to Oxidative States in the Cardiovascular System

**DOI:** 10.1155/2017/2712751

**Published:** 2017-05-14

**Authors:** Francesco Angelini, Francesca Pagano, Antonella Bordin, Marika Milan, Isotta Chimenti, Mariangela Peruzzi, Valentina Valenti, Antonino Marullo, Leonardo Schirone, Silvia Palmerio, Sebastiano Sciarretta, Colin E. Murdoch, Giacomo Frati, Elena De Falco

**Affiliations:** ^1^Department of Medical Surgical Sciences and Biotechnologies, Sapienza University of Rome, Latina, Italy; ^2^Institute of Cell Biology and Neurobiology-CNR, Monterotondo, Rome, Italy; ^3^Department of Radiology, Ospedale Pediatrico Bambino Gesù, Roma, Italy; ^4^Department of AngioCardioNeurology, IRCCS NeuroMed, 86077 Pozzilli, Italy; ^5^Aston Medical Research Institute, Aston Medical School, Aston University, Birmingham B4 7ET, UK

## Abstract

Oxidative states exert a significant influence on a wide range of biological and molecular processes and functions. When their balance is shifted towards enhanced amounts of free radicals, pathological phenomena can occur, as the generation of reactive oxygen species (ROS) in tissue microenvironment or in the systemic circulation can be detrimental. Epidemic chronic diseases of western societies, such as cardiovascular disease, obesity, and diabetes correlate with the imbalance of redox homeostasis. Current advances in our understanding of epigenetics have revealed a parallel scenario showing the influence of oxidative stress as a major regulator of epigenetic gene regulation via modification of DNA methylation, histones, and microRNAs. This has provided both the biological link and a potential molecular explanation between oxidative stress and cardiovascular/metabolic phenomena. Accordingly, in this review, we will provide current insights on the physiological and pathological impact of changes in oxidative states on cardiovascular disorders, by specifically focusing on the influence of epigenetic regulation. A special emphasis will highlight the effect on epigenetic regulation of human's current life habits, external and environmental factors, including food intake, tobacco, air pollution, and antioxidant-based approaches. Additionally, the strategy to quantify oxidative states in humans in order to determine which biological marker could best match a subject's profile will be discussed.

## 1. Introduction

The physiological cellular redox state can be defined as a fine and suitable balance achieved by the correct proportion of reactive oxygen species (ROS) within the cell microenvironment. The overall redox events are functional for living cells and equally involved in the cellular physiological maintenance and in response to a wide range of internal and external cues. Reactive oxygen species are intrinsic biological effectors of important mechanisms such as cell proliferation and differentiation, cell cycle progression, host defence, apoptosis, and migration [1–3]. This suggests their indispensable role in living cells.

Over the years, basic investigations on the pathophysiological role of cellular oxidative states on disease have been clinically confirmed. Specifically, a causal connection between enhanced ROS and increased risk of cardiovascular disease (CVD) has been largely demonstrated [4]. Endothelial dysfunction represents the hallmark of such event. Giving the endothelium's multiple roles in vascular homeostasis including tone, angiogenesis, remodelling, maintenance of blood fluidity, and as the first line of defence against systemic insults, the endothelium is more susceptible to ROS-based variations than other biological systems [5, 6]. In addition, increased oxidative states normally can coexist with concurrent enhanced levels of local or systemic inflammation. These two events are strongly linked, as they originate a reinforcing feedback loop, ensuing a potentiated effect. Interestingly, the pathophysiology of cardiac diseases based on imbalanced oxidative states can also occur as a consequence of hyperglycaemia, aging, atherosclerosis, or metabolic syndromes, known to negatively exacerbate vascular dysfunction [7]. Defined mechanisms underlying this effect have been currently reconsidered and explained by changes in functional behaviour of the genome induced by epigenetic mechanisms. The term “epigenetics” refers to modifications in the genome without any change in the primary DNA sequence information, thus only determining a different gene expression regulation of specific DNA regions. Accordingly, the affected regions can be either silenced or activated. Interestingly, epigenetic alterations can be inherited after cellular division, consequently representing one of the most significant known mechanisms by which living cells may respond to external stimuli and pass such adaptation to the following generation [8]. Either acute or chronic stress is able to modify the cellular epigenetic landscape, producing a wide and different range of effects according to each cell type. In the cardiovascular system, combinations of specific stress responses (hyperglycaemia, aging, and obesity) known to be directly related to CVD can influence progression of disease as they generate the “epigenetic memory” in cell populations [7, 9].

Importantly, an unbalanced redox state, caused either by increase of ROS and/or a reduced scavenging potential in cells (e.g., reduced antioxidants, scavenging enzyme lowered activity), deregulates several and diverse intracellular pathways related to “redox signalling.” In the heart, changes in the redox state of cardiac cells, including cardiac smooth muscle, vascular, and mesenchymal cells, represent a paramount source of stress, shown to trigger signalling cascades ultimately leading to changes in cell epigenetic states. Oxidative stress is able to unlock specific class IIa histone deacetylases (HDACs), whose role consist in repressing pathological gene expression [10]. Although epigenetic changes possess both reversible and irreversible features, nevertheless, they affect cardiac proliferation or generate specific cell type dysfunction and changes in cellular biology [7].

This has undoubtedly indicated that ROS production and epigenetic regulation represent an interconnected synergism, amplifying specific external factors towards the progression of pathological cardiac phenotypes. Moreover, life habits severely impact the link between ROS and epigenetic-mediated gene expression regulation, confirming that the control of cellular oxidative stress can be attained by simple lifestyle modifications [11, 12]. Accordingly, a beneficial potential of antioxidant-based therapies for CVD has been recently encouraged [13]. Yet, enhancing antioxidant system may in turn be detrimental [14] upsetting redox homeostasis. Nevertheless, besides the chemical synthetic molecules already available on the market targeting the binomial ROS epigenetic, great interest is shown on nonpharmacological natural antioxidants found in fruits and vegetables, significantly enriched in active molecules able to restore physiological redox states or simply acting as scavengers. Intriguingly, the food and recognized lifestyle habits typical of the Mediterranean area may be able to directly interfere with our genome through ROS production [15]. Researchers are aware that the precise mechanisms by which antioxidants act still remain to be fully determined, as well as the prediction of possible long-term clinical effects and limitations of this strategy. Additionally, it is unlikely that the sole restoration of physiological levels of ROS might ensure the reversion of cardiac symptoms. It seems more plausible that the management of the ROS/epigenetic axis could be useful to prime a preventive protection or to support current cardiac pharmacological therapies. More importantly, the lack of methodologies to properly quantify the oxidative state of a human subject represents a current challenge. Therefore, novel biological tests or markers to define a personal “ROS-based profile” are urgently required, in order to evaluate the efficacy of antioxidant-based strategies.

In this review, we aim to focus on the contribution of oxidative stress on cardiac pathophysiology with specific regard to the pathways by which ROS can effect epigenetic signals and thus cause the onset of pathological cardiovascular episodes. Additionally, the modality by which exogenous and environmental factors including aging, obesity, deficient dietary supplements, and smoking could impact the ROS-dependent epigenetics will be considered. Finally, novel frontiers to promote healthy lifespan, by employing a combination of antioxidant approaches and pharmacological interventions targeting epigenetic mechanisms, will be discussed.

## 2. The Oxidative Circuit in the Cardiovascular System: The Physiological Role Prior To Oxidative Stress

During embryogenesis, the heart is the first organ to be formed in order to sustain the development of the embryo. In the early phase of cardiogenesis, cardiomyocytes are in an active proliferative state, thus requiring an enhanced metabolic support to increase their biomass and to allow cell division. Mitochondria represent the power supply of all cells, by generating adenosine triphosphate (ATP), the cell energy currency, through the oxidative phosphorylation chain reaction. Interestingly, as cardiogenesis requires a metabolic overload, the mitochondrial structure transition from a round-fragmented shape, with an open permeability transition pore (mPTP), to an elongated structure with an enclosed mPTP normally occurs [16]. This physiological alteration is essential to improve the ATP production, suggesting that the redox state is fundamental for cardiomyocytes maturation in the early phase of cardiogenesis. A further critical effector during the development of the cardiovascular system is oxygen concentration. Indeed, the normal value of foetal arterial blood oxygen pressure is approximately 10 mmHg, meaning that low oxygen concentrations are sufficient to develop the cardiovascular circuit [17]. Specifically, low oxygen concentrations positively modulate hypoxia inducible factor 1*α* (HIF-1*α*) subunit activity. Once activated, HIF-1*α* transcriptionally fine tunes a panel of angiogenic, vasculogenic, and foetal heart remodelling genes, including the most relevant, vascular endothelial growth factor (VEGF). HIF-1*α* itself is under redox control with its activity regulated by S-glutathionyaltion, oxidative posttranslational modification [18]. Moreover, it regulates transcription of an anaerobic glycolysis-related set of genes, such as the glucose transporters, the lactate dehydrogenase, and pyruvate dehydrogenase kinase. This aspect is crucial, as the largest amount of the foetal cardiac ATP produced is derived from glycolysis and oxidation of carbohydrates. Interestingly, during childhood, this process is temporarily replaced by the catabolism of free fatty acids via *β*-oxidation.

Combined with the oxidative phosphorylation-based metabolic role, mitochondria in the cardiac tissue are the principal source of ROS [19]. Reactive oxygen species include reactive molecules and free radicals derived from molecular oxygen, such as superoxide anion (·O2^−^), hydrogen peroxide (H_2_O_2_), hydroxyl radical (·OH), and the reactive nitrogen species (RNS) nitric oxide (NO), and peroxynitrite (ONOO^−^). Over the years, ROS have been considered as damage-causing agents. To date, it has been demonstrated that subtoxic levels of O_2_^−^ and H_2_O_2_ can selectively interact with target molecules involved in cell signalling and regulation. Reactive oxygen species derived from cardiac mitochondria and NADPH oxidase (Nox) can increase HIF-1*α* expression and activity. Nox proteins are a family of seven membrane-associated multiunit enzymes that catalyse the reduction of molecular oxygen using NADPH as an electron donor. Only Nox1, 2, 4, and 5 are present in cardiovascular tissues [20], and Nox2 has been recently identified as one of the key mediators in mechano-chemotransduction during cardiac contraction [21, 22], whereas endothelial Nox2 is related to cardiac diastolic dysfunction [5, 6].

To date, strong evidence has supported the beneficial role of ROS in the adult cardiovascular system. For example, NO produced by the endothelial constitutive nitric oxide synthase (eNOS) is a key regulator of vascular tone [23]. Nitric oxide increases the cyclic guanosine monophosphate (cGMP) production and activates protein kinase G (PKG) and smooth muscle cell relaxation [24]. Likewise, hydrogen peroxide derived from endothelial Nox4 reduces blood pressure enhancing vasodilation [25]. Hydrogen peroxide mediates vasodilation, via modulating the cGMP pathway or directly acting on PKG [24]. Interestingly, cardiac-Nox4 protects against left ventricular hypertrophy through promoting angiogenesis [26]. In contrast, superoxide induced from Nox2 in cardiomyocytes contribute to remodelling postmyocardial infarction [27]. The role of ROS in vascular smooth muscle cell growth has been thoroughly investigated. Reactive oxygen species (in particular O_2_^−^ and H_2_O_2_) are produced in response to angiotensin II, platelet-derived growth factor (PDGF), epidermal growth factor (EGF), and fibroblast growth factor (FGF) ligand-receptor interactions. Once generated, ROS trigger the gene expression of cell proliferation-related genes, such as *c-myc* and *c-fos.*

Interestingly, ROS are able to exert a “contractile regulation” on cardiac muscle. Generally, cardiomyocyte contractile activity is based on the excitation-contraction coupling (ECC) process that transduces electrical excitation into contraction. During ECC, the membrane potential depolarization is followed by an inward current of Ca^2+^ caused by the opening of the voltage-gated calcium channels (LTCC), leading to a Ca^2+^-induced Ca^2+^ release by the ryanodine receptor (RyR) on the sarcoplasmic reticulum (SR). The ryanodine receptor family is composed of three isoforms, all exhibiting up to 89 cysteine residues per monomer [28]. The RyR2 is the main characterized redox-sensitive ion channels in the heart. Oxidizing conditions, and in particular NO, can increase the channel opening probability through the oxidation of SH groups, resulting in an increased Ca^2+^ loss from the SR [29]. Once released by RyR2, calcium activates myofilaments during systole by binding troponin C. If LTCC is inactivated and a K^+^ current is activated, repolarization occurs through the synergic regulation of various protein kinases and phosphatases. The SR Ca-ATPase SERCA2a is one of the principal effector of Ca^2+^ reuptake into the SR, which leads to relaxation by reducing cytosolic Ca^2+^. SERCA2a can undergo redox modification on its 25 cysteine residues, especially in the ATPase subunit [30]. In 2004, Cohen et al. demonstrated that low concentrations of NO-derived peroxynitrite (ONOO^−^) are able to directly increase SERCA2a activity via S-glutathionylation, promoting relaxation of cardiac muscle [31]. Likewise, a redox-activation of SERCA2b, the dominant isoform in vascular smooth muscle cells, with a consequent reduced intracellular Ca^2+^ level and vascular relaxation, has been reported [31]. ROS modulate cellular signalling through posttranslational modification on redox-sensitive cysteine thiols. Part of the cellular antioxidant pool include enzymes such as thioredoxin and glutaredoxin which catalyse the removal of oxidative posttranslational modifications. The preservation of redox homeostasis is essential, since increased glutaredoxin-1 can paradoxically attenuate postischemia neovascularisation [14]. Effective therapy will be needed to maintain the redox balance between ROS and antioxidants.

### 2.1. Oxidative Stress and Cardiovascular Disease Risk Factors

A large variety of risk factors significantly contributes to the onset of different types of cardiac conditions. Interestingly, most primary risk factors inducing an oxidative stress-dependent burden are related to life habits and environmental causes. However, they can be monitored and modified, theoretically allowing the potential restoring of the physiological cardiovascular clinical parameters. Among several cardiovascular dysfunction risk factors, aging represents a current debated exception, if considered as part of the normal process of life. Nevertheless, cardiovascular disorders are extremely frequent among elderly people and additional genetic, metabolic, pharmacological, or environmental variables may accelerate aging and consequently oxidative stress, leading to premature propensity to develop cardiovascular events.

In the light of this, in the second part of this review, we will discuss the genesis of oxidative stress induced in the setting of major CVD risk factors, including aging, smoking, and environmental pollution. Moreover, we will specifically focus on epigenetic determinants activated by oxidative stress and leading to cardiovascular disorder, either when occurring as primary pathological event or as secondary consequence of main contributor causes.

### 2.2. Aging

Aging is defined as a multifactorial process resulting in damage of molecules, cells, and tissues, leading to a reduced efficacy of functions with different pathophysiological consequences and a variety of clinical phenotypes [32, 33]. Cardiac fibrosis, ventricular hypertrophy, and myocyte enlargement with decreased cardiac mass represent the hallmark of aging in the heart [34], and likely induced by oxidative damage, among others. Accordingly, it has been demonstrated that an increased release of glutathione, together with a decreased release of oxidized glutathione, occurs in aged rat hearts. These alterations suggest that the metabolic and functional tolerance toward oxidative stress decreases with age, significantly enhancing the predisposition to CVD [35]. Exposure to highly reactive hydroxyl radicals may induce several alterations, including peroxidation of membrane lipids, inactivation of enzymes, and damage of nucleic acid base weights, which may all affect cell function. Furthermore, chronic exposure to oxidants, which exceed endogenous inactivation mechanisms, may result in a progressive accumulation of these biochemical alterations [36]. Additionally, the endogenous antioxidant system control, represented by the superoxide dismutase (SOD) and heat shock protein system (HSP), becomes weaker with aging, implying a lower resistance to ROS accumulation [37] and the progressive decline of protection from proteotoxicity in the mitochondria, apoptosis, and protein aggregation [38, 39]. Increasing age imbalances mitochondria biogenesis and mitophagy (i.e., autophagy of mytochondria), which are both essential for the maintenance of stress resistance and longevity [40].

An alternative mechanism involved in the aging process is represented by sirtuins, members of HDAC class III family, that, through mono-ADP-ribosyltransferase or deacetylase activity, are implicated in many biological processes, such as DNA repair, cell cycle regulation, apoptosis, and gene expression [41]. Nuclear SIRT1 mediates antioxidant stress responses by activating the FOXO subfamily of the forkhead family of transcription factors [42]. FOXO subfamily is involved in a wide range of crucial cellular processes regulating stress resistance, metabolism, cell cycle arrest, apoptosis, and protein homeostasis, affecting autophagy and proteasome-related gene expression [43]. SIRT1 has been related also to mitochondrial biogenesis, acting on the peroxisome proliferator activated receptor γ coactivator 1*α* (PGC-1*α*) [44] and HIF-1*α* [45]. Other sirtuins have been detected in mammalian mitochondria, such as SIRT3 and SIRT5 [46]. SIRT3 regulates several enzymes, such as SOD2 and mitochondrial isocitrate dehydrogenase (IDH2), that are critical in maintaining cellular ROS levels [47]. SIRT5 is able to bind and activate the carbamoyl phosphate synthetase (CPS1), which is required for removing ammonia generated by amino acid catabolism [48] and regulates also ammonia-induced autophagy [49]. These protective effects are crucial because ammonia can increase the production of ROS, inducing the mitochondrial permeability transition (MPT), known as a common pathway leading to oxidative stress-induced apoptosis also in cardiomyocytes [48, 50]. Overall, these evidences indicate that sirtuins are important therapeutic targets potentially able to delay the progression of aging-related pathologies, such as type 2 diabetes or Alzheimer's disease, and perhaps to extend lifespan. Notably, sirtuin expression and activity levels decline with age in several tissues, implying that induction of long-term enhancement of sirtuin activity represents an interesting challenge for the scientific society [51].

### 2.3. Hyperglycaemia and Obesity

The physiological events discussed can shift to pathological states in the presence of clinical insults, such as metabolic disease, obesity, and diabetes [52–55], as well as exogenous and environmental factors (pollution and tobacco). Lifestyle habits are able to affect aging, through exacerbating ineffective protection from oxidative stress both in adult and in young subjects. Obesity exerts a great impact on ROS regulation, as it is defined as chronic low-grade systemic inflammation associated with structural and functional changes in the perivascular adipose tissue (PVAT), appropriately considered as an endocrine tissue. This inflammatory state leads inevitably to vascular endothelial and smooth muscle cell dysfunction, thus increasing cardiovascular risk. All these alterations represent the foundation of enhanced release of ROS, vascular tone, and proinflammatory factors. It has been demonstrated that high fat depots within the body result in an enhancement of energy accumulation, dysfunction of mitochondrial oxidation, and oxidative stress [56]. Recently, Costa et al. demonstrated that PVAT anticontractile effects are mediated by ROS directly generated from mitochondrial metabolism [57]. In fact, PVAT-derived tumor necrosis factor alpha (TNF-*α*), known as a mediator of adipose tissue inflammation, can induce mitochondrial oxidative stress in the form of increased production of^.^O_2_^−^ and consequently conversion in H_2_O_2_ and modulation of the RhoA/ROCK-pathway, a key player in numerous smooth muscle cell functions, including contractility [58]. This evidence has suggested that ROS generation could represent a fundamental mechanism involved in obesity-associated PVAT vascular dysfunction [59]. Moreover, systemic alterations strictly associated to ROS have been observed in obese patients. According to this scenario, high levels of oxidative stress can be detected both in overweight young individuals and in obese adults, where an elevated baseline of proinflammatory mediators and of the biomarker F2-isoprostanes is observed, respectively [60–62]. Interestingly, F2-isoprostanes are directly correlated with HDL-cholesterol levels, adipocytokines, measures of adiposity, and total body and abdominal subcutaneous fat [63]. Despite unequivocal evidence demonstrating a link between obesity and oxidative stress, specific prognostic markers for monitoring the evolution of clinical obesity are still to be identified, although some of them have been successfully employed to predict the progression of metabolic CVD in overweight and obese people [64].

One of the most significant medical risk factor of obesity is the insulin resistance state, phenotypically exhibited as type 2 diabetes, where cells develop a low sensitivity to the hormone, thus causing hyperglycaemia. Obesity-associated inflammation, that initially affects adipose tissue and liver, plays a key role in this phenomenon as it inhibits several insulin-associated signalling pathways, such as insulin receptor substrate 1 (IRS-1) [65], and it increases plasma-free fatty acid (FFA), thus stimulating lipolysis [66]. In this pathological scenario, oxidative stress could be considered as both an effector and a consequence, considering that mitochondrial function significantly contributes to insulin secretion from pancreatic *β*-cells [67]. Accordingly, mitochondrial increased ROS production promotes accumulation of FFA [68], subsequently increasing the serine/threonine kinase activity (PKC) that inactivates IRSs [69]. An alternative mechanism of oxidative stress induction has been described, showing that the increased presence of FFA may enhance ROS concentration by downregulating antioxidant enzyme activity, such as peroxisomes and microsomes [70].

Defects in mitochondrial fatty acid oxidation could be involved in insulin resistance development, as it induces increased fatty acid metabolites able to block insulin signalling [68]. These derangements underlie the known direct detrimental effects of diabetes on cardiac structure and function, which may contribute to the development of heart failure and ultimately to death in diabetic patients [71]. The high ratio of circulating FFA typically detected in the obese and diabetic population contributes to store fatty acids in the cardiac tissue and to negatively regulate mitochondria-based cell metabolism, where fatty acid oxidation instead of glucose oxidation occurs, jeopardizing ATP production and causing higher oxygen consumption [72, 73]. This significant metabolic change leads to altered energy availability in cardiomyocytes, to the generation of lipotoxicity because of the accumulation of ceramide and diacylglycerol (DAG) inside the myocardium, and to a gradual decrease of cardiac function [74].

Moreover, SIRT1 is equally and physiologically involved in the regulation of insulin secretion from pancreatic *β*-cells, enhancing ATP production and leading to cell membrane depolarization and Ca^2+^-dependent insulin exocytosis [75]. Interestingly, SIRT1 also exerts a protective effect from oxidative stress in pancreatic *β*-cells by suppressing the proinflammatory nuclear factor-kappaB (NF-*κ*B) signalling [76] and the expression of tyrosine phosphatase-1B, which in turn negatively regulates the insulin receptor (IR) and IRS-1 [77]. During a lifespan, SIRT1 activity decreases, but adipose tissue cells of obese subjects already exhibit a reduction, confirming the exacerbating effects of metabolic diseases on physiological states [78].

### 2.4. Exogenous and Environmental Factors

Beyond pathophysiology-dependent oxidative stress, it is noteworthy to consider that the organism is constantly exposed to exogenous factors known to induce specific stress responses. Among the wide range of environmental elements in the western society, those related to ecology and lifestyle, including pollution, smoking, and alcohol, have received a special focus. Free radicals or substances able to trigger free radical reactions, contained for example in the form of pollutants in the atmosphere, are usually derived from either car exhaust pipes and fumes or factories' waste products. It has been reported that the exposure to this kind of agents and in particular to pollutants increases the risk for CVD. Mechanisms underlying such phenomenon include the enhancement of the oxidative stress, inflammatory response, and susceptibility to coagulation/thrombosis/atherosclerosis [79]. Besides, in 2010, during the first scientific statement on “Air Pollution and Cardiovascular Disease”, the American Heart Association has drawn the attention on a novel air pollutant, named the particulate matter (PM), currently considered as a major risk factor for cardiovascular morbidity and mortality [80]. Furthermore, particles of <0.1 *μ*m diameter (the so-called ultrafine particles) are able to infiltrate the tissue very easily, showing a preferential localization in the mitochondria. Similar to other pollutants, the ultrafine particles can induce ROS generation and, subsequently, oxidative stress in epithelial and macrophage cells [81].

Likewise, cigarettes are considered one of the major lifestyle-related risk factors for health. Accordingly, it has been demonstrated that smoking induces multiple side effects such as inflammation, oxidative stress, energy metabolism abnormalities, gap junction remodelling, hypertrophy, and impaired angiogenesis, thus leading to myocardial damage [82]. Interestingly, tobacco increases Nox activity (known to exert a role in cardiac remodelling) and induces depletion of antioxidant enzymes, causing oxidative damage and exacerbating cardiac fibrosis, myocyte hypertrophy, and systolic dysfunction [83]. Furthermore, smokers exhibit high levels of circulating inflammatory cytokines, such as TNF-*α* or IFNs, involved in cardiac remodelling, cell death, and ROS production [84]. Of note, it has been recently demonstrated that electronic cigarettes (E-cigarettes), a modern and technological surrogate of traditional tobacco cigarettes, have unfavourable effects on markers of oxidative stress and FMD after single use [85]. The smoking-related health risk is consistently aggravated in the presence of obesity. Recently, it has been confirmed that this binomial is responsible for inducing cardiomyocyte apoptosis by both increasing oxidative stress and by mechanisms of inactivation of the AMPK pathway [86].

Alcohol can also affect the cardiovascular system in a dose-dependent manner, causing a lower ejection fraction and left ventricular hypertrophy [87, 88]. It has been demonstrated that, in the presence of an alcoholic cardiomyopathy, a reduce activity of respiratory enzymes, lactate dehydrogenase, and fatty acid oxidases, combined with an increased alcohol dehydrogenase activity may occur. The oxidation of the accumulated acetaldehyde promotes ROS generation, resulting in organelle damage, lipid peroxidation, and autophagy [89]. In alcoholic patients, ROS are able to directly affect atrial fibrillation by exacerbating oxidative modifications in the myofibrillar creatine kinase and thus inducing an increase in contractile force [90, 91]. Interestingly, physical exercise exerts a dual influence on oxidative states. Specifically, a regular and moderate training stimulates the endogenous antioxidant system and the associated adaptive responses, thus preserving the correct muscle redox balance [92]. Likewise, an exhaustive aerobic and anaerobic physical exercise can generate ROS overproduction, leading to oxidative stress-related tissue damages and impaired muscle contractility. Of note, the final effect on the redox balance strictly depends on age, sex, and training level, as well as individual susceptibility to oxidative stress injury, which is known to be determined by genetic and lifestyle factors [93].

### 2.5. Oxidative Stress and Epigenetics in the Cardiovascular System and Disease Risk Factors: A Worthy Link to Explore

**Figure 1:**
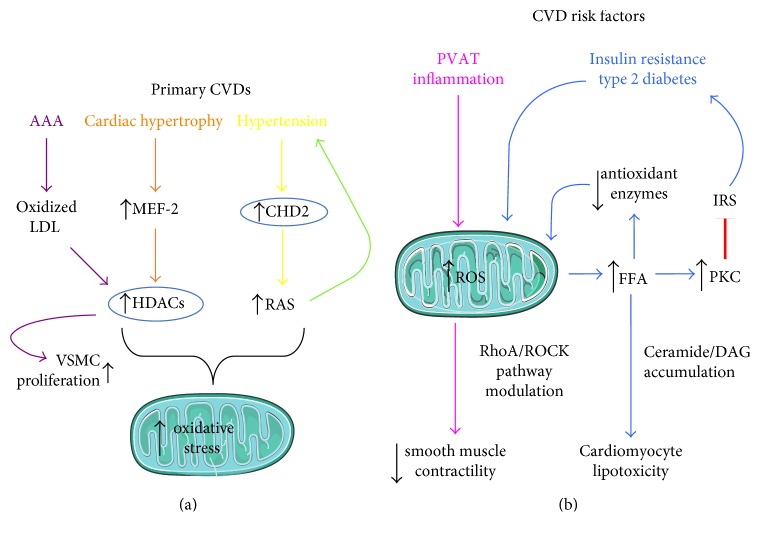
(a, b) Main oxidative stress-dependent epigenetic alterations and molecular mechanisms in primary CVD (a) and CVD risk factors (b). AAA: abdominal aortic aneurism; LDL: low-density lipoprotein; VSMCs: vascular smooth muscle cells; MEF-2: members of enhancer factor-2; HDACs: histone deacetylases; CHD2: chromodomain-helicase-DNA-binding protein 2; ROS: reactive oxygen species; FFA: free fatty acids; IRS: insulin receptor substrate; PKC: serine/threonine kinase activity; PVAT: perivascular adipose tissue; RAS: renin-angiotensin system; DAG: diacylglycerol.

It is extremely challenging to unequivocally identify oxidative stress-dependent epigenetic determinants specifically involved in the onset of primary cardiovascular disorders and also to discriminate from those derived by risk factors (a main summary of epigenetic mechanisms highlighted in this paragraph is displayed in Figures 1(a) and 1(b)).

The epigenetic modifications in the heart represent a key step in the remodelling process, where both acetylations and methylations target downstream mediators. Importantly, substantial cardiac stress directly acts on class IIa HDACs, involved in repressing main pathological genes [10]. Oxidative stress can disable this activity through the phosphorylation-independent nuclear export mechanism of HDACs [10].

Pathological cardiac hypertrophy is strictly driven by the overexpression of members of enhancer factor-2 (MEF-2) family, which are known to be regulated by class IIa HDACs [94]. Alternative epigenetic acetylation determinants targeting HDAC4 and influencing cardiac hypertrophy have been described in combination with enhanced nuclear oxidative stress caused by Nox4 [94]. In addition to this scenario, several biological methylation modifications employ the conversion of S-adenosyl methionine, a known antioxidant molecule, to S-adenosyl homocysteine, whose increased levels, found in patients with CVD [95], are able to directly activate the DNA methyltransferases [96]. Intriguingly, this phenomenon can be reinforced by a parallel oxidative stress-dependent epigenetic mechanism (mitoepigenetic) in the mitochondria, main producers of considerable amount of ROS following an ischemic insult. The mitochondrial DNA methyltransferases activity is less sustained compared to that in the nucleus, but equally enhanced by high levels of homocysteine [97]. From a biological and clinical standpoint, the hypomethylation negatively drives the angiogenic and endothelial gene expression, resulting in vascular complications. The mitochondria dynamic also plays a key role in heart failure, as their damage via enhanced ROS runs parallel to free calcium in the cardiomyocytes and volume overload [98].

Intriguingly, the hemodynamic stress clearly impacts gene expression reprogramming via oxidative stress. Accordingly, a direct association between oxidative stress-dependent epigenetic pathways and the trimethylation of histones H3K4 and H3K9, resulting in decreased left ventricular ejection, a hallmark of chronic heart failure, has been demonstrated [99, 100].

Likewise, patients with vascular disorders, such as abdominal aortic aneurism (AAA), display increased expression of HDAC 1, 2, 4, and 7 in aortic tissues with respect to healthy subjects, with concomitant changes in DNA methylation of inflammatory cytokine promoters [101]. Interestingly, a main role of ROS in exacerbating the systemic inflammatory landscape in AAA has been hypothesized, given the ability of ROS to directly activate histone acetyltransferases in several cell types [102]. A similar alteration of the muscle-specific histone acetylation profile has been also reported in vascular smooth muscle cells (VSMCs), in presence of high levels of oxidized LDL able to combine HDAC2 and 5-mediated hypoacetylated states with the reactivation of embryonic transcription factors (i.e., Krüppel-like factor-4) permissive to VSMCs uncontrolled proliferation [7]. A similar effect can be observed in the lung, where arterial smooth muscle cell proliferation and inhibition of apoptosis are both enhanced by epigenetic attenuation of the mitochondrial superoxide dismutase (SOD2) [103]. A further mechanism has been described in hypertension. The renin-angiotensin system (RAS) is a major controller of systemic blood pressure. A sustained activation of RAS observed in hypertension is able to increase ROS levels through NADPH activation and its catalytic subunits [6, 104]. The associated upstream epigenetic signal has been linked to the chromodomain-helicase-DNA-binding protein 2 (CHD2), an enzyme acting on histone deacetylation and chromatin remodelling [105] and able to overactivate the transcriptional site of RAS [106].

The impact of oxidative stress on the cardiac genetic reprogramming via “pathological” alterations of the epigenetic machinery is even more substantial if we consider cardiovascular risk factors strictly associated to enhanced ROS levels (diabetes, obesity, aging, and life habits). Certainly, one of the most peculiar and oxidative stress-related epigenetic modification can be found in type 2 diabetes, a major risk factor for cardiovascular disorders. Diabetic patients affected by CVD display abnormal genomic cytosine-guanine (CpG) islands associated with multiple oxidative stress markers. The CpG sites contain regulatory regions of the genome, suggesting a potentially direct role of oxidative stress caused by hyperglycaemia, or in presence of CVD, on methylation of specific genes and their expression [107]. Interestingly, patients with acute ischemic stroke exhibit enhanced levels of systemic 5-methylcytosine [96] with additional alterations in nucleobases induced by peroxides [108]. Importantly, it has been observed that diabetes can generate long-term clinical complications even when physiological glucose levels are restored by pharmacological therapies or thorough modifications in patients' dietary habits and exercise frequency. This phenomenon is well known as metabolic memory [109]. Metabolic memory has been implicated in the persistence of epigenetically induced aberrant expression of fibrotic, antioxidant, and inflammatory genes in many cell types, including vascular smooth muscle and endothelial cells [110]. For example, hyperglycaemia can lead to increased levels of mitochondrial ROS in aortic endothelial cells, inducing long-lasting monomethylation of the histone H3 at the lysine 4 (H3K4) and lysine 9 demethylation (H3K9) sites in the proximal promoter of the NF-*κ*B subunit p65. The methylation of both histones 3 and 4 can act synergistically, leading to the aberrant activation of the downstream proinflammatory pathway [110, 111].

Noncoding RNAs, and specifically microRNAs (miRNA), known as alternative mediators of epigenetic mechanisms, have been recently linked to the establishment and maintenance of metabolic memory. MicroRNAs can directly affect cellular epigenetic states, by modulating the expression of several epigenetic factors [112]. Some miRNAs can also directly interact with gene promoters and therefore stimulate or repress their activity in a process acknowledged as RNA activation (RNAa) [113]. In addition, different from the protein complexes responsible for DNA and chromatin modifications, miRNAs can be released into the bloodstream and captured by different cell types. This phenomenon allows to deregulate the expression of selected miRNAs and to perpetuate the effect from one cell to another. Notably, hyperglycaemia, oxidative stress, and inflammation can all modify the profile of circulating miRNAs and affect the transcriptional program of vascular smooth muscle and endothelial cells, contributing to the exacerbation of vascular complications [109]. Some miRNAs seem to play a key role upon high glucose levels. Diabetic murine vascular smooth muscle cells show increased expression of miR-125b able to target the Suv39h1 gene, responsible of enhanced expression of inflammatory mediators [114]. MiR-125b has also been shown to be directly responsive to H_2_O_2_ in treated human keratinocytes (HaCaT) [115], strengthening the strict relationship between oxidative stress and changes in microRNA activity.

Growing evidence is currently supporting a significant relationship between aging-associated diseases and epigenetic changes [116]. Some effects caused by aging are directly generated by alterations in gene expression or activity of DNA and chromatin-modifying enzymes. Histone deacetylates, such as SIRT-1, have been shown to decrease in protein level during physiological aging, leading to age-related increase in the acetylation of lysine 16 of histone H4 (H4K16). A global hypomethylation of an aged genome has been associated with decreased activity of DNA methylation enzymes [9]. The epigenetic effects of oxidative stress associated to aging have been well described in endothelial cells, where specific inhibition of SIRT-1 generates a senescent phenotype in the HUVEC model system, involving p53 gene acetylation followed by growth arrest [117]. SIRT-1 activity inhibition during senescence can also be induced by upregulation of miR-217, which targets SIRT-1 3′UTR and induces its posttranscriptional silencing in different endothelial systems, including HUVECs and human aortic and coronary artery endothelial cells [118]. More recently, miR-34a has been described to have a similar role in mediating cardiac, endothelial, and endothelial progenitor cell senescence [119]. A direct effect of oxidative stress on microRNA expression has been shown on endothelial cells, where H_2_O_2_ treatment increases miR-200 family members, triggering downregulation of the zinc finger homeobox 1 (Zeb1) transcription factor. These molecular alterations lead to senescence and apoptosis [120].

Interestingly, longevity is also correlated with metabolic rates. For instance, production of *α*-ketoglutarate via oxidative deamination of glutamate is able to influence chromatin demethylation [121]. In particular, ROS production is inversely linked with lifespan. Therefore, ideal strategies aiming at reducing the metabolic rate may possess “rejuvenating features.” Gene profiling analysis in mice subjected to caloric restriction has demonstrated that low caloric intake can modify the aging rate and reduce the accumulation of detrimental macromolecules related to oxidation processes, leading to a 20% longer lifespan [122, 123]. This effect has been considered protective for telomere length, but against cardiovascular impairment associated to aging and stress [124, 125]. Epigenetic mechanisms involving sirtuins, and specifically SIRT-1, have been related to this phenomenon.

As already mentioned, another important clinical disease is obesity, a complex condition determined by a simultaneous interaction between genetic features and environmental factors. Recently, it has been undoubtedly highlighted that epigenetic changes imprint obesity. Accordingly, obese adolescents display DNA methylation alterations in blood cells [126]. Adipose tissue is currently considered an important endocrine organ, secreting various hormones, cytokines (adipocytokines), and free fatty acids (FFAs) [127]. Reactive oxygen species production in obese patients is generated by a chronic inflammatory status promoted by FFA oxidation in the liver and perpetuated by macrophages and monocytes, which are constantly stimulated by circulating inflammatory cytokines [128]. It has been shown that stearoyl-CoA desaturase 1 (SCD1), an enzyme involved in monounsaturated fatty acids generation, directly affects inflammatory mediators production in adipocytes through DNA methylation [129]. In fact, changes in SCD1 expression are inversely correlated with global DNA methylation changes, where modifications in CpG methylation of interleukin-10 receptor a (IL10ra), interleukin-4 receptor a (IL4ra), interleukin-6 signal transducer (IL6st), and transforming growth factor *β*1 (TGF*β*1) genes are changed by SCD1 overexpression [130]. Functional studies have also revealed a direct association between chromatin-modifying enzymes and obesity. Genetic knockout of the Jumonji C-domain containing protein (Jhdm2a), a H3K9 demethylase, is able to increase mice susceptibility to obesity by abnormal fat deposition and hyperlipidaemia, likely due to PPAR*α* expression decrease in skeletal muscles and brown fat [131].

Obesity-related inflammatory, oxidative, adipogenetic mechanisms and insulin signalling can be also concomitant to miRNAs deregulated expression. The heme oxygenase-1 (HO-1) has cytoprotective effects on CVD, and its activity is reduced in obese mice. Increasing HO-1 activity has shown functional benefits against insulin resistance and compensatory hyperinsulinemia in animals. MiR-155, miR-183, and miR-872, which are reduced by insulin treatment of adipocytes, regulate HO-1 activity and levels after insulin administration [132]. The same miRNAs also contribute to inflammatory cytokines production, oxidative damage, and apoptosis.

Epigenetic variations can be also induced by exogenous factors, such as cigarette smoking. Several components in cigarette smoking can directly alter both methylation and acetylation balances of chromatin, leading to deregulation of specific genes involved in inflammation [133]. In particular, specific effects on a decreased activity of HDAC2 has been reported, as well as complete inactivation and proteasomal degradation in the lung cells and macrophages in vivo [134, 135]. A recent study in vitro on first branchial arch-derived cells has showed that the exposure to cigarette smoking extract (CSE) induces global DNA hypomethylation due to proteasomal degradation of two main DNA methyltransferases (DNMT-1 and DNMT-3a) and two methyl CpG-binding proteins, MeCP-2 and MBD-3 [136].

## 3. Biological Markers of ROS

The quantification of circulating ROS through specific biomarkers could represent a promising mean not only to detect pathological cardiovascular processes but also to predict and monitor patients' responses to therapeutic treatments. The identification of which type of molecule would best suit this purpose is still challenging, as ROS are extremely unstable, and several physiological process may generate diverse oxidative states and/or be differentially influenced by them. Besides, circulating amounts of ROS might not entirely reflect the local scenario at cellular or tissue level. Therefore, an indirect measure of ROS could also be informative. Accordingly, a panel of biomarkers of oxidative stress is currently available, and it includes molecules that can be modified by interacting with ROS themselves. Intriguingly, as DNA, lipids, phospholipids, proteins, and carbohydrates are selective targets of ROS, they can all mirror the level of oxidative states in patients [137]. Alternatively, the estimation of the antioxidant counterpart might be relevant at least to understand an unexpected occurrence of an unbalanced redox state. Folic acid, contained in vegetables, is a suitable example to describe how natural oxidants might potentially function as a litmus test of the cardiovascular state in humans. In fact, folic acid is able to decrease the circulating levels of homocysteine in the blood, whose amount is strictly interdependent to enhanced risks of vascular and cardiac disorders [138]. Notably, some molecules, such as isoprostanes (IsoPs) and malondialdehyde (MDA), both generated by peroxidation of lipids, also hold a prognostic cardiovascular value. The levels of F_2_-IsoPs, prostaglandin-like compounds, are elevated in conjunction with specific cardiovascular risk factors, including cigarette smoking, diabetes mellitus, and myocardial ischemia/reperfusion events. Similarly, isoprostanes are also generated during human atherosclerotic lesions formation [139] and therefore involved in vasoconstriction, platelet aggregation, and proliferation of vascular smooth muscle cells [61, 140]. To date, antioxidant therapies based on the employment of vitamin C and/or E integration have been shown to decrease the levels of IsoPs [141], although these observations are not confirmed. Differently, MDA, generated in vivo by the peroxidation of polyunsaturated fatty acid, can be easily quantified in plasma by a colorimetric assay based on its reaction with thiobarbituric acid (TBA), generating a final product defined as TBARS (TBA reacting substances) [137]. These latter have been found decreased in diabetic rats by the antioxidants alpha-lipoic acid and aminoguanidine [142–144]. Consequently, this assay represents a useful biological test of oxidative stress associated with CVD.

“Alternative” free radicals such as nitric oxide (NO), one of the most powerful vasodilator, could be a further interesting oxidative biological marker. Nitric oxide is known to alter both structural integrity and catalytic activity of several proteins by tyrosine nitration and cysteine S-nitrosylation and glutathionylation. In aging rat hearts, nitration partially inhibits sarcoendoplasmatic reticulum Ca^2+^ ATPase 2a (SERCA2a) activity [145]. Despite this, due to its reduced half-life, whether or not the quantification of NO levels could accurately provide any relevant clinical information is still to be fully verified. In a recent work, Di Gioia et al. have observed that iron supplementation was able to restore the detrimental effects of the oxidative/nitrosative damage involved in SERCA2a-related diabetic heart complication [146]. In line with this scenario, other studies have reported that the increase in nitrotyrosine content in diabetic mice may lead to an enhanced susceptibility to myocardial infarction [147]. On a molecular level, ROS-responsive genes such as nuclear factor (erythyroid-derived 2)-like2 (Nrf-2) and peroxisome proliferator-activated receptor gamma coactivator 1-alpha (PCG-1*α*) may be used as biomarkers to assess cardiovascular redox status [148, 149].

The identification of oxidative biomarkers is not only relevant for prognostic reasons but also to understand the complex and assorted response to pharmacological treatments in human subjects. Direct antioxidants, such as vitamins C and E, beta carotene, and glutathione, are able to control ROS flow and prevent the accumulation of their electron excited metabolites [150]. Pharmacotherapies based on angiotensin receptor blockers (ARB) [151, 152], statins, HMG-CoA reductase inhibitor [153], or *β*-adrenergic receptor blockers [154, 155], have shown compelling effects on markers of oxidative stress. Therefore, ROS-associated biomarkers may be positive indicators of the outcome of a specific therapy.

## 4. Antioxidant Approach by Natural Agents: The Road to Detect and to Modify Our Own Oxidative State

The emerging role of redox mechanisms regulating epigenetic pathways in a synergistic fashion has led to the development of new drugs with antioxidant activity, in order to target specific methylated sequences of DNA, histone modifications, and noncoding RNAs, all strictly related to CVD. Oxidative stress produces DNA methylation changes [156], and several inhibitors of this phenomenon, such as the synthetic 5-aza-2-deoxycytidine (a demethylation agent [157]), may act as cardiac protectors in the presence of a cardiac pathological condition. Recently, a special attention has been dedicated to those nonpharmacological natural antioxidants and active ingredients contained in fruits and vegetables, including polyphenols, cocoa, and folic acid [158–160]. A wide number of studies are demonstrating how edible plants intrinsically exert the highest antioxidant effect in humans, providing the rationale of their daily consumption in a balanced diet plan. Molecular mechanisms and biological advantages derived from natural agents are still to be clarified; however, these significant experimental observations have led to the “nutrigenomic” perspective, referring to the ability of food to interfere with the genome, in other words, a sort of epigenetic of the food.

Notably, the Mediterranean dietary habit stands out as it is mainly based on natural antioxidant ingredients [161]. Its combination with an active lifestyle has been successfully associated with reduced rates of CVD in the corresponding populations [162]. It is still arduous to comprehend whether the beneficial outcome of natural antioxidants in plant-derived food on the cardiovascular circuit is more related to a systemic rebalance of the oxidative states rather than a selective effect. However, strong evidence is supporting this latter hypothesis. For instance, apocynin (4-hydroxy-3-methoxy-acetophenone), extracted from the root of *Picrorhiza kurroa* (Kutki), is a well-recognized antioxidant [33], described to prevent excessive collagen deposition and cardiomyocyte apoptosis in rats [163]. Olive oil, enriched with polyphenols, is clearly recognized to decrease the levels of oxidized-LDL and to preserve haemostasis, blood pressure, and physiological levels of insulin by positively interfering with the cardiovascular gene expression profile [164, 165].

Curcumin, a member of the ginger family, is another example since it is able to epigenetically modulate the activity of HATs and HDACs [166]. Specifically, it increases the levels of HDAC2 by preventing its degradation under oxidative and nitrosative stress [133, 134]. Curcumin also exerts several cardioprotective effects: it activates SIRT1 in cardiomyocytes, inducing the antiapoptotic gene Bcl-2 and downregulating proapoptotic genes such as Bax, beclin-1, and BNIP3 [167]; it suppresses profibrotic activity in cardiac fibroblasts [168]; and it reduces oxidative stress-induced mitochondrial damage in heart cells [169]. In a mouse model of cardiac hypertrophy, curcumin can suppress the acetylase p300/CBP, consequently decreasing both inflammatory and fibrotic responses [170, 171].

Moreover, Cui et al. have recently demonstrated protective effects of *α*-lipoic acid on male mice subjected to high-fat diet. Α-lipoic acid restores the redox balance of T cells reducing significantly ROS accumulation through Nrf2 nuclear translocation and upregulation of Nrf2 target genes as Ho-1 and Prdx1 [172].

Intriguingly, two beverages enriched in natural polyphenols, that is, red wine and green tea, which have been consumed through the centuries in the Mediterranean area and Asian countries, respectively, are also providing evidence of health benefits. Epicatechins (flavonoid family), the major component of green tea (but also of cocoa and grapes), have been demonstrated to restore the expression of antioxidant enzymes through demethylation of glutathione-S-transferase P1 (GSTP1) promoter and the inhibition of DNMT [173]. An indirect positive effect of the consumption of green tea has been also observed due of its ability to decrease oxidized LDL, a biological marker of obesity [174]. Epicatechins contained also in cocoa would attenuate inflammation in human monocytes subjected to hyperglycaemia by rebalancing appropriate levels of acetylations and methylations of histone 3 [175]. More importantly, epicatechins have been described to diminish endothelial activation induced by activated platelets [176]. Resveratrol is another interesting natural molecule, also abundantly present in peanuts, grapes, and other berries [177], that can act as a potent antioxidant at lower concentration compared to other agents in a dose-dependent fashion [178]. Resveratrol increases the expression of antioxidant enzymes, such as glutathione peroxidase, glutathione S-transferase, and glutathione reductase [179], and acts by scavenging free radicals as well [180]. Notably, resveratrol is able to regulate gene expression by epigenetic activity. Accordingly, both natural and synthetic resveratrol derivatives, such as resveratrol-salicylate, can concurrently act as DNA methylation blockers and antioxidant partners by inhibiting DNMT3 and DNMT3b enzymes and by decreasing the expression of myeloperoxidase (MPO), respectively [181]. Resveratrol is also involved in the induction of SIRT1, known to upregulate eNOS expression via deacetylation of FOXO1 [182]. The activation of FOXO1 results in increased expression of antioxidant genes, such as manganese superoxide dismutase (Mn-SOD) and catalase [183]. Interestingly, the beneficial effects of the resveratrol would also encompass the vascular compartment. Resveratrol upregulates the expression of HO-1 in artery endothelial and myocardial cells, reducing the infarcted area and preserving cardiac function in a rat model of acute myocardial infarction [184, 185]. Moreover, in a recent work, Shen et al. have reported that resveratrol restores the cardiovascular repair capacity via peroxisome proliferator-activated receptor-γ (PPAR-γ) and HO-1 signalling pathways and prevents senescence of endothelial progenitor cells (EPCs), known to be severely affected by oxidative stress [33, 186]. To date, several preclinical and clinical trials are confirming the efficacy of resveratrol in treating cardiovascular disorders when administered for a short period [187]. Accordingly, low doses (8 mg/kg) of resveratrol for one year significantly reduce the number of cardiac risk factors [188].

In addition to natural compounds, researchers are exploiting many alternative molecules, attempting to solve the dilemma of whether epigenetic alterations in the cardiovascular system induced by oxidative stress may be counteracted. Accordingly, HDAC inhibitors are a chemically synthesized broad-spectrum class of molecules successfully used for treating several pathologies, including heart disorders [189]. HDACis can be categorized into six distinct groups: short-chain fatty acids, hydroxamates, cyclic tetrapeptides, benzamides, electrophilic ketones, and miscellaneous [190, 191]. Successful examples of clinically effective HDACis are tributyrin, valproic acid (VPA), trichostatin A (TSA), and vorinostat (SAHA), whose administration reduces significantly cardiac fibrosis in a murine model of acute ischemia by left descending coronary artery ligation [192–194]. TSA improves myocardial remodelling after ischemia-reperfusion injury by inhibiting HDAC4 in cardiomyocytes [192]. Notably, Aune et al. have demonstrated that entinostat (MS-275), an inhibitor of class I HDAC, exerts a powerful regenerative effect on restoring contractile heart function, combined with increased antioxidant properties when compared to several nonselective and selective HDACis [195]. To date, the employment of HDACis in cardiovascular disorders is suggesting their clinical validation even in presence of anti-cancer therapies that may act as pro-oxidant agents [196]. In particular, it has been demonstrated that phenylbutyrate (PBA) protects cardiac tissue from oxidative stress generated by the anticancer agent Adriamycin through the modulation of manganese superoxide dismutase (MnSOD) [197].

Alternative epigenetic therapies include administration of miRNA inhibitors and mimics. In fact, ncRNA dysregulations are associated with cardiovascular and metabolic diseases [198, 199]. Circulating miRNAs such as miR-126, miR-17, miR-145, and miR-222 are currently considered indicators of cardiovascular risk [200], as their levels in body fluids or plasma reflect the evolution of cardiovascular disorders, including myocardial infarction, heart failure, diabetes mellitus, stroke, and acute pulmonary embolism [201, 202]. Some miRNAs could also possess prognostic value. In a recent study, Coskunpinar et al. have observed increased expression levels of miR-221-3p in acute myocardial infarction [203]. MicroRNAs are also able to regulate the expression of genes controlling the generation of ROS, known to contribute to fibrotic accumulation. Therefore, therapeutic approaches targeting miRNAs involved in regulating redox balance involved in fibrosis may represent a novel strategy for drug development [204]. Understanding how coenzyme Q10 (CoQ10) supplementation can help diabetic patients reveals low glutaredoxin-1 levels after treatment [205].

## 5. Conclusions

A healthy and functional oxidative circuit is indispensable to preserve physiological homeostasis of the cardiovascular system, but it could also reflect pathological states when altered. Consequently, this aspect of the redox state combined with both experimental observations and preclinical results highlighting that a strict relationship between unbalanced ROS production and altered epigenetic modifications in cardiovascular disorders, could reveal as prognostic value.

A current challenge is represented by the difficulty to distinguish oxidative stress-dependent epigenetic patterns induced by primary CVD from those induced by risk factors. Several epigenetic determinants directly caused by ROS are still to be discovered, as well as the role of the histonic regulation beyond the solely methylation. Accordingly, a discrete number of recent reports have confirmed the clinical efficacy of distinct classes of HDAC inhibitors in CVD [10]. Notably, oxidative stress forms are hereditable, likely implying defined hereditable epigenetic changes. This scenario could represent a solid and sufficient background to accumulate over time further alterations in the genome, that in presence of environmental factors prepare for CVD. On the other hand, epigenetic mechanisms are reversible. Therefore, we expect to extend this hypothesis also to those alterations strictly related to oxidative stress. This could support an alternative therapeutic vision of the management of patients in presence of a cardiovascular insult: oxidative states can be monitored by intervening on the genome and vice versa by means of synthetic or natural epigenetic modulators, antioxidants agents, and healthy life habits.

**Figure 2:**
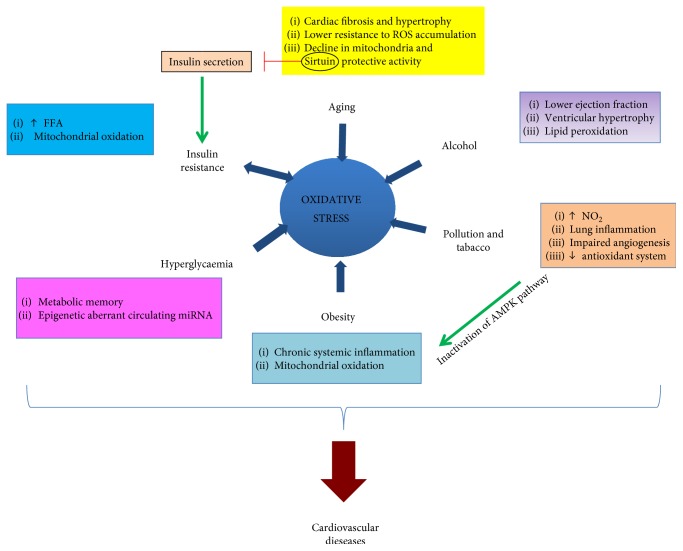
A schematic summary of the major factors determining alterations of the physiological redox states.

Despite this, additional drawbacks are predictable. Cellular oxidative balances and epigenetic profiles are likely to be dissimilar between patients. Moreover, life habits and environmental factors also play a key role in this phenomenon, further exacerbating differences between subjects and undoubtedly impacting the binomial relationship between ROS and epigenetics (Figure 2). Researchers are also seeking for novel biomarkers by which both physiological and pathological oxidative states could be finely defined, but also exploited as eligible prognostic criteria. Some drugs targeting epigenetic signalling may help in the future, but only after clearly identifying potential tissue or systemic epigenetic consequent alterations.

We envision that in the future a multiple rather than a single medical approach based on patient's oxidative profile should be encouraged.
